# Yttrium-90 distribution following radiosynoviorthesis of the knee joint in rheumatoid arthritis patients: a SPECT/CT study

**DOI:** 10.1007/s12149-014-0827-8

**Published:** 2014-03-05

**Authors:** A. Bielińska, M. Korkosz, J. Gąsowski, M. Tomaszuk, A. Staszczak-Sowa, B. Kwaśny-Krochin, M. Buziak-Bereza, A. Hubalewska-Dydejczyk, T. Grodzicki

**Affiliations:** 1 Department of Rheumatology and Balneology, Jagiellonian University Medical College, Sniadeckich 10, 31-501 Kraków, Poland; 2 Division of Rheumatology, Department of Internal Medicine and Gerontology, Jagiellonian University Medical College, Kraków, Poland; 3 Department of Internal Medicine and Gerontology, Jagiellonian University Medical College, Kraków, Poland; 4 Nuclear Medicine Unit, Department of Endocrinology, University Hospital in Krakow, Kraków, Poland; 5 Nuclear Medicine Unit, Department of Endocrinology, Jagiellonian University Medical College, Kraków, Poland

**Keywords:** Rheumatoid arthritis, Radiosynoviorthesis, Yttrium-90 isotope, SPECT/CT

## Abstract

**Objective:**

To examine yttrium-90 distribution 1 and 72 h following its injection into a knee joint in patients with rheumatoid arthritis (RA).

**Methods:**

In 14 RA patients we injected yttrium-90 into the affected knee joint using lateral approach. To assess the radioisotope distribution in the joint, the superimposed sequential SPECT and CT imaging was performed 1 and 72 h after the injection. We analyzed the percentage of radioisotope distribution in three predefined compartments of the knee joint (lower, upper medial, upper lateral).

**Results:**

After 1 and 72 h, the mean percentage distributions were, respectively, 7.14 and 23.07 % in lower; 21.42 and 15.38 % in upper medial, and 71.42 and 61.53 % in upper lateral compartment. The percentage of isotope deposition did not change significantly with time in any of the compartments (all *p* > 0.26). The deposition of isotope, both at 1 and 72 h, was significantly greater in upper lateral compartment, where the injection was performed, than in all other compartments (all *p* < 0.05).

**Conclusions:**

Using the SPECT/CT hybrid method, we proved that the majority of isotope is located at the compartment adjacent to the injection. Two injections targeting different compartments might improve the clinical efficacy of the procedure.

## Introduction

Radiosynoviorthesis (RSO) is a safe and effective therapeutic modality for synovitis in inflammatory arthritis [1–3]. It was developed as an alternative to or preceding the surgical synovectomy [4]. The main indication is a chronic synovitis of affected knee in rheumatoid arthritis (RA). Yttrium-90 (^90^Y), a radionuclide with an intense β-emission, is most widely used for the treatment of knee joint. Efficacy of the RSO is estimated at 60–80 % [3]. However, the accurate prediction of the efficacy of RSO is difficult due to patient-related factors such as the type, course, and inflammatory activity within the joint [2, 5]. On average, penetration of radiation following RSO approximates 10 mm [6, 7]. Therefore, the efficacy of RSO largely depends on the radionuclide distribution within the joint.

Single photon emission computed tomography (SPECT/CT) is a novel hybrid imaging method that combines functional data from SPECT and anatomic data from CT, sequentially acquired during single examination [8]. SPECT creates computer-generated images of radionuclide distribution, while CT enables 3D anatomical images of joint.

The majority of published studies assessed the distribution of radionuclide using gamma camera/SPECT imaging yielding imprecise information which does not reflect the uptake of radionuclide into the anatomic compartments of the knee. Nevertheless, the gamma camera imaging provided an evidence that even high degree of standardization of the procedure, including site of injection, needle gouge does not prevent unpredictable distribution patterns [9]. Moreover, there are no guidelines for follow-up time needed to assess RSO efficacy.

The aim of this study was to assess, using SPECT/CT, the yttrium-90 radionuclide distribution 1 and 72 h following its injection into RA affected knee joint.

## Methods

We prospectively enrolled 14 consecutive patients diagnosed with RA according to 1987 American College of Rheumatology criteria [10]. Knee synovitis had to be present despite treatment with disease-modifying antirheumatic drugs and at least one intra-articular administration of long acting steroid. We excluded patients with ruptured popliteal cyst, pregnant or breast-feeding or unable to give informed consent.

## Procedures

### Radiosynoviorthesis

Radiosynoviortheses with beta emitting radionuclide injected into the knee joint were performed according to European Association of Nuclear Medicine guidelines [3]. The yttrium-90 (^90^Y), Colloid CIS bio international colloid suspension for local injection (IBA Molecular, France), with mean activity of 220 MBq, was administered into each predetermined knee. Arthrocentesis of the knee with 1.2 mm-gauge needle was carried out from lateral approach by experienced rheumatologist. One needle was used for all intraarticular procedures. Effusion, if present, was aspirated before radionuclide injection which was immediately followed by intraarticular administration of 7 mg of betamethasone. After steroid injection the knee was passively bent several times and splinted for 48 h.

### SPECT/CT assessment

In order to assess the radioisotope distribution in the joint space and to find the possible leakage of radioisotope outside the joint, the SPECT/CT examinations were performed. A hybrid system Symbia TruePoint T16 camera (Siemens) with parallel, medium energy collimators was used. The yttrium-90 (^90^Y) tracer was injected in each target knee. All patients underwent SPECT/CT examination of the knee 1 and 72 h after the injection of the radionuclide. Settings of SPECT scan were as follows: 180° orbit for each head, step and shoot mode, 40 images, at 32 s per view, 128 × 128 matrix. The acquired data were reconstructed using the ordered subset expectation maximization (OSEM) FLASH 3D iterative reconstruction method with eight subsets and ten iterations. Setting of CT part of the study was as follows: 130 kV, effective mA s 190 with CAREDose4D system, slice thickness 5.0 mm, acq. 16 × 1.2 mm and reconstruction increment 3.0 mm with B50s moderate kernel shaped. Each SPECT/CT was evaluated on an ongoing basis by the medical physicist. In case of suspicion of leakage of the isotope beyond the joint, assessment was extended to regional lymph nodes.

**Fig. 1:**
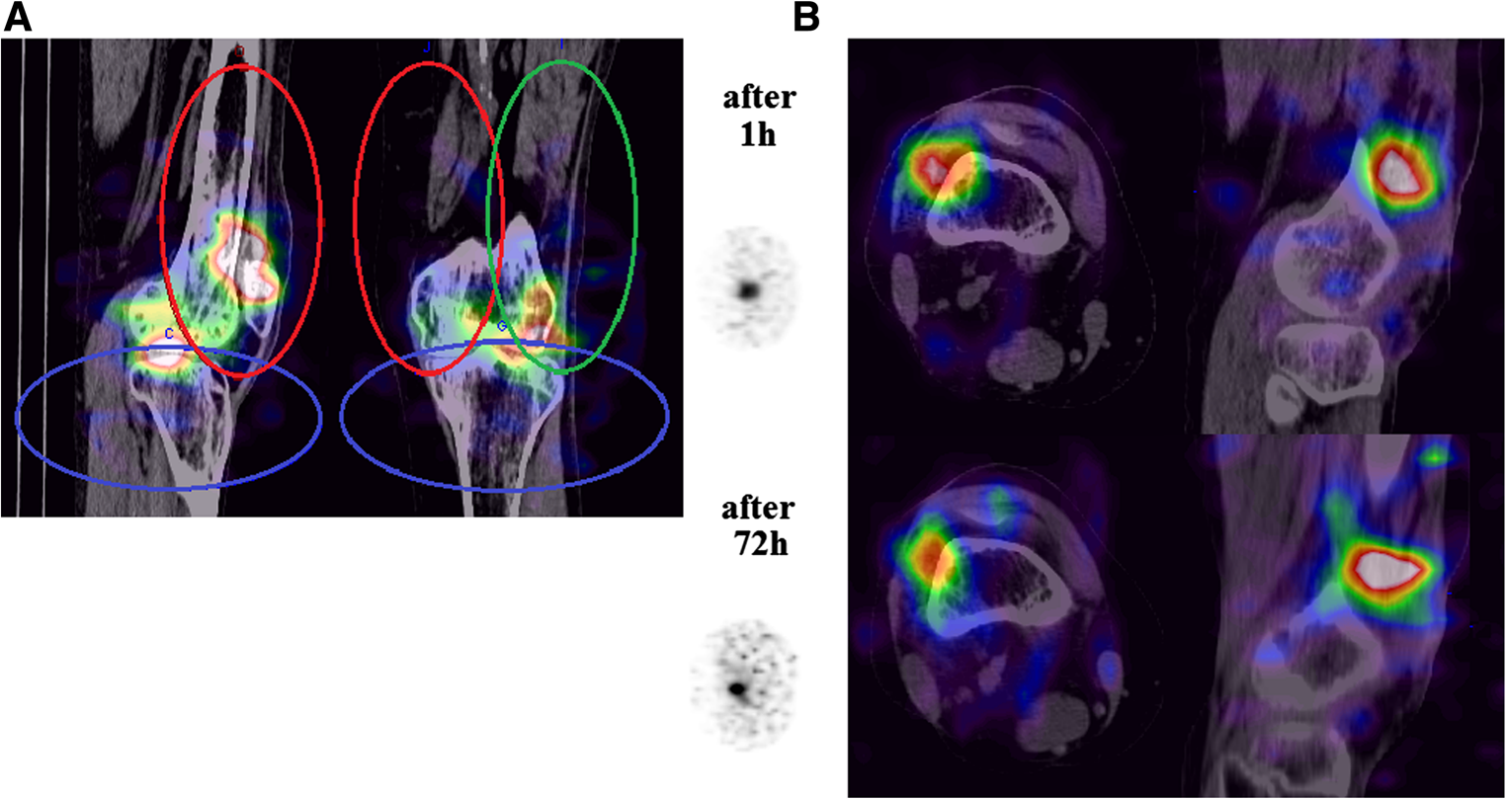
Radioisotope distribution within the knee joint. **a** Ellipsoid 3D predefined regions of interest: upper lateral (*green*), upper medial (*red*) and lower (*blue*) compartments for isotope distribution measurement. **b** The example of the average distribution of yttrium-90 in the knee joint 1 and 72 h after procedure (color figure online)

Knee joint was divided into three ellipsoid 3D shaped regions of interest (ROI) according to the predefined anatomical compartments: upper lateral, upper medial and lower (Fig. 1a). Percentage analysis of radioisotope distribution in three compartments was performed during SPECT/CT examinations (each in 72 h apart). The total amount of injected yttrium-90 (^90^Y) was considered as 100 %. The number of counts in each ROI was evaluated using manufacturer provided volumetric analysis. The percentage of uptake [%] in each region was assessed according to the formula: [%] = number of counts in the region/the sum of counts from all regions ×100.

The study was approved by the local bioethics committee, and written informed consent was obtained prior to the enrollment.

### Statistical analysis

Data were analysed using SAS 9.2 software (SAS Institute Inc. Cary, NC, USA). First we established the normality of frequency distributions. Then, we used the Student’s *t* test and univariate ANOVA model to check for the between-compartment, and within-compartment time-related differences, respectively.

## Results

**Fig. 2:**
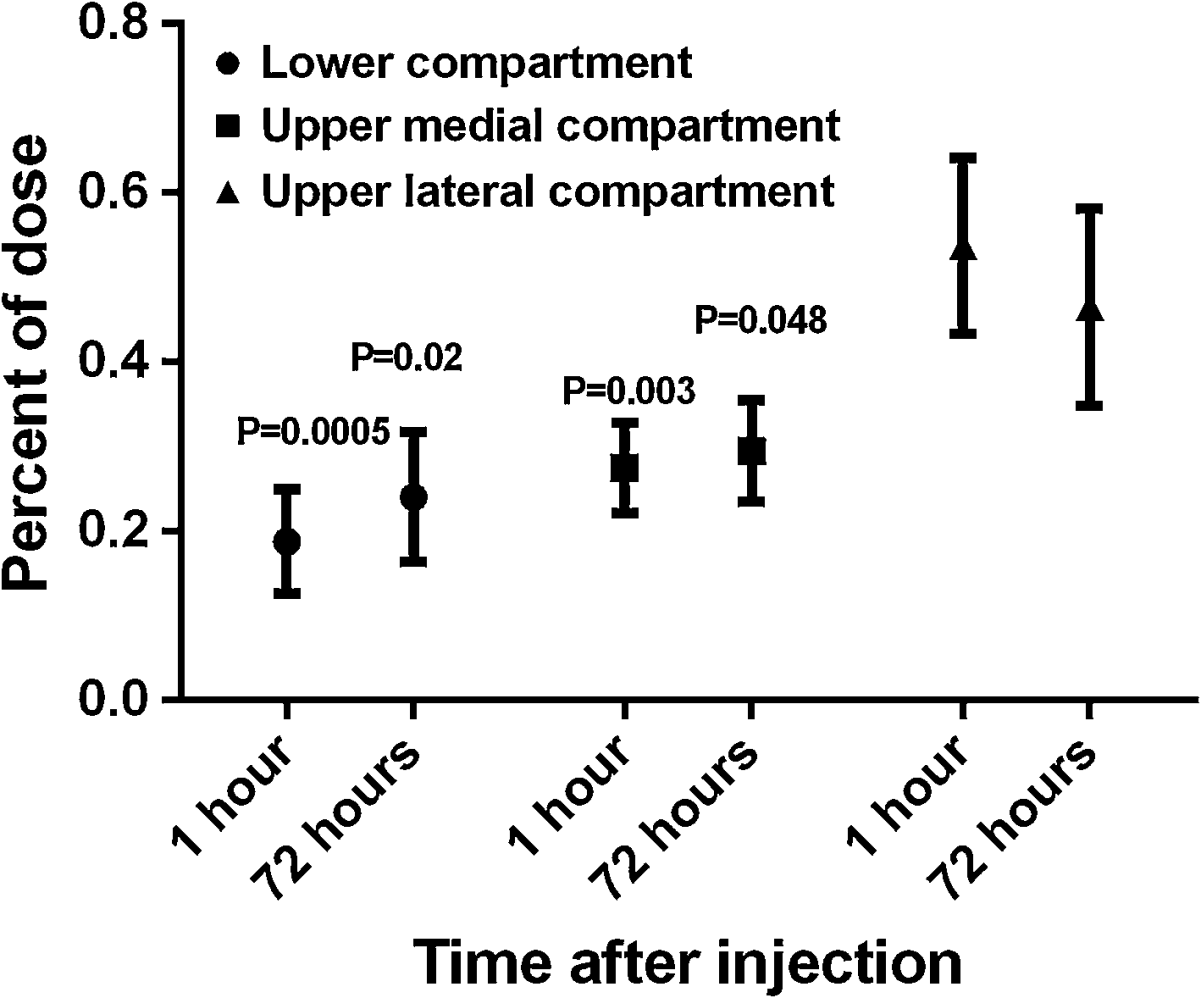
The distribution of yttrium-90 in three knee compartments 1 and 72 h after radiosynoviorthesis. *P* values for comparisons between upper lateral compartment (injection site) and two other compartments, 1 and 72 h after procedure, respectively. The *error bars* are 95 % confidence intervals

Mean ± SD age of patients was 53 ± 12 years, with mean RA duration prior to the study of 5.46 ± 3.31 years. The mean percentage distributions of the isotope within compartments, after 1 and 72 h, respectively, were as follows: in the lower compartment 7.14 and 23.07 %; in the upper medial compartment 21.42 and 15.38 %; in the upper lateral compartment 71.42 and 61.53 %. After 1 h of injection, we found significant differences of uptake between compartments, with the greatest percentage of isotope deposition at the upper lateral compartment which was adjacent to the injection site (all *p* < 0.003), (Table 1). This did not change materially 72 h post procedure, with significant difference between the compartment of the injection site and each of the remaining two (Fig. 2). The percentage of isotope deposition did not change significantly with time at any of the compartments (all *p* > 0.26) (Table 1).

**Table 1:** The radioisotope colloid distribution into the knee joint space examined by SPECT/CT method

Patient no.	Lower compartment				Upper medial compartment				Upper lateral compartment			
	T0 (after 1 h)		T1 (after 72 h)		T0 (after 1 h)		T1 (after 72 h)		T0 (after 1 h)		T1 (after 72 h)	
	Measured value	Ratio (%)	Measured value	Ratio (%)	Measured value	Ratio (%)	Measured value	Ratio (%)	Measured value	Ratio (%)	Measured value	Ratio (%)
1	187,082.0	17.3	160,291.3	34.0	484,612.4	44.9	166,620.9	35.4	40,858.3	37.8	144,356.6	30.6
2	217,307.1	21.0	127,637.3	28.5	335,644.8	32.5	194,931.8	43.6	480,038.4	46.5	124,793.7	27.9
3	245,048.1	27.2	144,419.4	38.2	343,020.1	38.0	112,091.4	29.6	313,543.0	34.8	121,849.3	32.2
4	64,939.4	7.5	20,950.9	13.7	186,765.1	21.6	26,292.3	17.2	613,708.4	70.9	105,246.2	69.0
5	58,825.2	10.3	64,441.6	17.6	133,025.3	23.3	150,761.8	41.1	378,311.1	66.3	151,367.9	41.3
6	80,293.5	31.2	276,710.3	30.5	96,891.2	37.6	307,239.7	33.8	80,179.1	31.1	324,050.1	35.7
7	73,337.4	28.2	69,108.2	10.1	71,726.6	27.5	279,255.6	40.7	115,310.6	44.3	337,686.4	49.2
8	28,775.6	7.8	21,597.8	9.2	39,720.1	10.7	31,132.0	13.2	302,540.8	81.5	182,240.8	77.6
9	114,714.3	8.0			361,218.5	26.0			905,109.1	66.0		
10	72,946.2	9.3	78,084.6	11.4	133,805.9	17.1	141,477.4	20.7	575,082.2	73.6	464,146.0	67.9
11	93,577.8	37.5	44,320.3	42.7	75,823.4	30.4	33,047.0	31.8	79,817.9	32.0	26,398.4	25.4
12	64,144.3	29.6	36,393.9	37.0	63,848.2	29.5	30,732.5	31.2	88,605.2	40.9	31,319.1	31.8
13	17,597.1	6.6	8,672.9	7.5	44,348.0	16.5	18,703.9	16.7	205,999.4	76.9	88,372.5	76.3
14	45,041.6	21.9	24,868.4	32.2	58,956.9	28.7	21,640.9	28.0	101,222.8	49.3	30,667.1	39.7

## Discussion

We found that the distribution of yttrium-90 (^90^Y) isotope following its intra-articular injection into the knee joint in RA patients highly depends on the site of injection. The deposition was greatest in the upper lateral compartment into which the isotope was injected, and this did not change significantly 72 h after the procedure (Fig. 2). The innovative approach of our study was to use the SPECT/CT which enabled accurate anatomical localization of the deposited isotope (Fig. 1b).

To the best of our knowledge, so far only two studies assessed the distribution of an isotope, following the injection into a knee. Jahangier et al. studied 69 patients in whom RSO was performed [9]. They used the gamma-camera based detection of the distribution of the isotope, which does not permit the accurate anatomical visualization. They found that in the majority of patients (54 %) the deposition was diffuse. However, only 28 % of their group were RA patients. Furthermore, they did not standardize the time of the assessment, which was performed immediately or 24 h after injection, making it impossible to study the time-related changes in isotope distribution. Likewise, Jahangier et al. failed to report whether the deposition was related to the site of injection. We used a hybrid method enabling accurate anatomical visualization of the isotope within a knee which enabled us to prove that the isotope is deposited predominantly at the site of injection, which in our study was standardized, and that deposition does not change after 72 h of follow-up.

Vuorela et al. did a feasibility study on six RA patients [6]. In order to localize synovitis they administered a ^99m^Tc-immunoglobulin [^99m^Tc-HIG] labeled anti-synovial antibody. Then they treated the patients with 166 Holmium ferric hydroxide macroaggregates (^166^Ho-FHMA), and reassessed the SPECT/MRI. The authors concluded that the hybrid method using MRI and SPECT is feasible, and that the administration of an isotope-labeled antibody can help localize synovitis. However, the patient was exposed to two sources of radiation including one injected systemically (500–550 MBq), and one injected intra-articularly (0.9–1.0 GBq), and further used gadolinium-enhanced MRI scanning. We also used the gadolinium-enhanced MRI to localize synovitis, however, we did not use the isotope-labeled anti-synovial antibodies. The SPECT/CT required single source of radiation of 220 MBq, and the CT equipment we used featured a low-energy scanner.

Our results must be considered within the context of the study’s limitations. Slight overlapping of ellipsoids is visible on presented 2D figure (Fig. 1a) but it mostly covers bone regions or other anatomical structures outside the joint cavity. The overlap is estimated to be less than 10 % but might have certain impact on our results.

In conclusion, our data are the first to show, using the SPECT/CT hybrid method of visualization, the precise anatomical distribution of injected isotope, and the fact that the distribution does not change over the standardized, 72 h time of imaging follow-up. The fact that the majority of isotope, as opposed to findings by Jahangier et al. is located close to the site of injection, inclines us to hypothesize that two injections at two different sites targeting different knee compartments might improve the distribution of isotope. Alternatively, a larger volume of radioisotope colloid suspension might enhance the distribution of yttrium-90 within the knee joint and augment the clinical outcome of the procedure. However, further studies are needed to assess these hypotheses.
